# Reducing Antimicrobial Use and Dependence in Livestock Production Systems: A Social and Economic Sciences Perspective on an Interdisciplinary Approach

**DOI:** 10.3389/fvets.2021.584593

**Published:** 2021-03-18

**Authors:** Fanny Baudoin, Henk Hogeveen, Erwin Wauters

**Affiliations:** ^1^Social Sciences Unit, Flanders Research Institute of Agriculture, Fisheries and Food, Merelbeke, Belgium; ^2^Business Economics Group, Wageningen University & Research, Wageningen, Netherlands

**Keywords:** antimicrobial resistance, antimicrobial use, livestock production, systems thinking, behavioral change, interdisciplinary research

## Abstract

**Objective:** In livestock production, antimicrobial resistance (AMR) is considered an externality as it is the undesired result of preventive and curative antimicrobial use. To address this biosocial issue, our objective is to present an approach based on interdisciplinary research to develop strategies and policies that aim to contain AMR.

**Method:** To do so, we addressed three fundamental questions on which control policies and strategies for agricultural pollution problems are centered in the light of AMR. To ensure the technical, economic, behavioral and political feasibility of the developed measures, we demonstrated the usefulness of systemic approaches to define who, what and how to target by considering the complexity in which the ultimate decision-maker is embedded. We then define how voluntary or compulsory behavioral change can be achieved via five routes, introducing a clear taxonomy for AMR Interventions. Finally, we present three criteria for ex-ante analysis and ex-post evaluation of policies and strategies.

**Conclusion:** Interdisciplinary systemic approaches enable the development of AMR policies and strategies that are technically, politically, economically and, last but not least, behaviorally feasible by allowing the identification of (a) all actors influencing AMU in livestock production, (b) power relations between these actors, (c) adequate regulatory and intervention bases, (d) what behavioral change strategy to use, (e) whom should implement this, as well as the cost-effective assessment of combinations of interventions. Unfortunately, AMR policies and strategies are often investigated within different disciplines and not in a holistic and systemic way, which is why we advocate for more interdisciplinary work and discuss opportunities for further research.

## Introduction

For the two past decades, concerns regarding antimicrobial use in farm animals grew considerably due to the growing prevalence of antimicrobial resistance (AMR) and the way this affects human health. AMR is a natural process that results from the ability of microorganisms to quickly adapt to changing conditions. Indeed, the appearance of rare and advantageous mutations that neutralize the effects of antimicrobials is inevitable in large and dense microbial communities and the rapid generation times allow these mutations to quickly become prevalent in growing communities (1). Additionally, bacteria have the capacity to exchange mobile genetic elements, including resistance genes, via horizontal gene transfer within and between bacterial species, further enhancing their ability to adapt (1).

But while AMR is a natural phenomenon, its increasing prevalence is most certainly not. In fact, it is fueled by anthropogenic factors such as the intensive clinical and agricultural use of antimicrobials worldwide, the growth of the world's human population, changes in human lifestyle (e.g., increased urbanization, migration and travel), and misconceptions and malpractices regarding antimicrobial use (AMU) (1). Over time, this increasing prevalence is predicted to have a significant impact on global health and wealth by potentially causing up to 10 million deaths each year, at a cumulative cost of $100 trillion to global economic output by 2050 (2). To further contextualize this, the World Bank Group estimated that reductions in annual global GDP due to AMR (ranging between 1.1 and 3.8%) may be comparable to the losses caused by the 2008–2009 financial crisis, with the difference that the economic damage would continue for decades and would mostly affect low-income countries (3).

To contain this serious threat to global health and wealth, the 194 member states of the World Health Organization (WHO) endorsed a global action plan (GAP) in 2015 and committed to establishing national action plans (NAPs) based on the “One Health” approach, which recognizes the interaction between human health, animal health and the environment (4). By 2018, 60% of Member States declared having a NAP in place and 33% reported that they were in the process of developing one (5). This global attempt to contain AMR with a One Health approach seems timely, since it was estimated that by 2030, antimicrobial consumption, which has now been repeatedly associated with AMR (6, 7), would increase by 67% in livestock (8), by 33% in aquaculture (9) and by 15, 32 or 202% in humans, depending on the scenario (10).

In the European Union, this political will to contain AMR has led to a European strengthening of the response to AMR with the development of an EU One Health action plan against AMR and new EU regulations on veterinary medicines [Regulation (EU) 2019/6] and medicated feed [Regulation (EU) 2019/4] (11). In practice, the Member States' efforts to reduce AMU in veterinary medicine, and mainly in animal husbandry, resulted in a 32,5% decrease in sales of veterinary antimicrobial medicinal products between 2011 and 2017 (12). While this seems to be a good start to achieve a more sustainable use of antimicrobials in European livestock production, there are still challenges ahead. Further efforts will be needed to reach the European Commission's target of a 50% reduction of antimicrobial sales for farmed animals and aquaculture by 2030, as set out in the recently adopted “Farm to fork” strategy. The degree of effort required to achieve this target is also likely to differ between Member States, given that large variations in AMU trends have been observed between European countries, with some using 136 times as many antimicrobials for the rearing of food-producing animals (12). Finally, in addition to significant differences in national AMU trends, monitoring at farm level also revealed variations between farms, species, and production cycles (13, 14), further complicating the picture.

To address the challenges posed by antimicrobial resistance, countries have been advised to invest in AMR containment (3) through AMR surveillance and by curbing the prevalence of antimicrobial resistance via optimal antimicrobial prescription and use in both human and veterinary medicine. Regarding the latter, the institutionalization of AMU as well as the reduction of antimicrobial dependence is necessary in order to achieve a sustainable use of antimicrobials. In livestock production, antibiotics play a crucial role since they are not only a therapeutic but also an economic asset. The preventive use of antimicrobials to treat at-risk herds or animals (prophylaxis) as well as clinically healthy animals sharing premises with symptomatic animals (metaphylaxis) allows the limitation of economic risks and labor costs (15, 16). Outside of Europe and the USA, antimicrobials are also used as feed additives, which are thought to improve animal growth, feed conversion and yield and allow farmers to keep pace with the demand for meat while lowering the prices (17). To reduce this reliance on antimicrobials, the focus is often on information and technological innovations as vaccination and alternatives to antimicrobials. But while investments in (therapeutic) innovations are foreseen in the GAP, and presumably the NAPs based thereon, the promise of new technologies might not be enough. In fact, therapeutic alternatives to antimicrobials are currently not sufficiently developed in order to effectively replace antimicrobials (18). Considerable investment in research and development will be needed, which means that these options will not be widely available in the coming years. Moreover, it is very likely that such options will offer short-term solutions since we are engaged in an infectious arm's race with microbes that always find a way to accommodate to new therapeutics. In this regard, Smith (19, 20) esteems that the vision for AMR control is currently focused on technological and biomedical innovations, the benefits of which could be short-lived if our society remains heavily dependent on antibiotics. In addition, there is no guarantee that alternatives will be immediately adopted by farmers, as it was the case for the live oral Lawsonia vaccine in pigs, that was not widely used despite positive results (18). Studies cut across many disciplines have shown that the adoption of new technologies by farmers can be influenced by numerous factors, e.g., environmental factors such as land use (21) and land characteristics (22); personal features such as age, human capital or risk preferences (23); economic attributes such as market intervention by regulators (24) and costs of acquiring the technology (25); extension services (22) as well as cultural and social factors including social identity (26), social networks (27, 28) and peer group influence (29). It is therefore clear that farmers' behavior is embedded in both biophysical and social landscapes (30) and that decision-making processes are complex and context dependent. In addition to this, other actors in the social landscape may also indirectly influence farmers' behavior by voluntarily or involuntarily creating physical (e.g., land appropriation) or social structures (e.g., norms) that restrict, or enlarge, farmers' opportunity space (30). To better understand farmers' behavior while considering the systemic complexity in which it is embedded, several frameworks and systemic approaches have been developed (30–32) with the hope that this would help design research that represents farmer's behavior more realistically and that it would lead to the development of more effective sustainable agriculture policies.

In this respect, our objective is to add to an interdisciplinary research agenda by providing a perspective on strategies for reducing the dependence on AMU and the threat of AMR from a social science and economic point of view. This perspective was inspired by how social scientists and economists contributed to environmental policies (33). We discuss how knowledge about farmers' behavior and the system in which they operate can contribute to answering three central questions for the development of policies and strategies and can provide a clear taxonomy of AMR interventions in livestock production. To better illustrate this, we also provide examples of existing policies and strategies to address antimicrobial use and dependence. Next, we present three criteria for ex-ante analysis and ex-post evaluation of these policies and strategies and, finally, we discuss the importance of an interdisciplinary approach to get insights in farmers' behavior and the system they are embedded in upon introducing research opportunities.

## Three Fundamental Questions for Defining Policies and Strategies to Mitigate AMR in Livestock

Since AMR in livestock and agricultural pollution are both externalities, the design of policies and control strategies for the latter might also be useful for the former. We therefore used three fundamental questions on which the design of policies and control strategies for agricultural environmental pollution is centered (34) to develop an approach to design new policies and strategies to mitigate AMR in livestock. The first fundamental question is: *who* among those who play a role in the production of an externality should be targeted. The second question aims to determine the basis for measuring effectiveness or, in other words, *what* variable(s) control policies and strategies wish to change. Finally, the third question is *how* to target, i.e., by what mechanism(s) the intended actors and bases should be targeted.

### Identifying Key Actors in Antimicrobial Decision Systems

To reduce antimicrobial use and dependence in livestock production, it is necessary that farmers, as ultimate users, and veterinarians, as antibiotics prescriber, change their behavior. It is thus only logical that policies and strategies target them. This is the case in Denmark, where veterinarians do not have the right to sell veterinary drugs (35) and pig farmers need to remain under set antimicrobial use thresholds if, according to the yellow card initiative, they do not want to face legislative implications, including a reduction of the stocking density of animals (36).

However, the solution to the question “who to target” does not need to be limited to the actual users of the antimicrobials. Indeed, when dealing with externalities, it has been suggested that, in addition to the actual source, others could be targeted (34). This idea has been reinforced by systemic approaches that suggest that there are many more actors who have an influence on how food is being produced and that often, farmers are end-of-pipe decision makers largely influenced by the practices and demands of other actors in the system (30, 37). In this regard, value chain approaches for the analysis of animal health systems grew in popularity as they allow for the analysis of the different actors involved, their roles, and the interactions between the actors as well as how this influences practices (38). Since value chains are in turn embedded in a bigger biological, social, economic, and regulatory context, analytical frameworks have been developed to study such big and complex systems. For example, Lamprinopoulou et al. (39) developed a framework to analyze Agricultural Innovation Systems (AIS), which consists of innovation processes that encompass all type of knowledge that all actors in an agricultural system demand and provide, as well as the interaction between these actors. The framework allows to define the functions and structures, i.e., identification and classification of actors, of an AIS and to assess how, at a micro level, systemic failures may affect the contribution of actors to the fulfillment of the functions of the AIS. Moreover, the functioning of the entire system is also explored by investigating if basic structural components and functions are sufficiently coordinated, aligned and harmonized. This approach was further used by Rojo Gimeno et al. (37) to comprehensively depict swine health systems by identifying key actors and their functions as well as merits and failures at micro and macro level that impact functions. A shortcoming of such approaches might be that the natural environment is not taken into account. To fill this gap, Hagedorn et al. (40, 41) developed and an analytical framework to analyze nature-related transactions, which has later been applied to agricultural soil conservation by Prager (31). Here, the interdependencies between ecological and social systems are taken into account by considering the biophysical characteristics of soil and the related farming practices that contribute to soil degradation, as well as all the actors, policies, institutions, instruments, and governances structures that may influence these.

**Figure 1:**
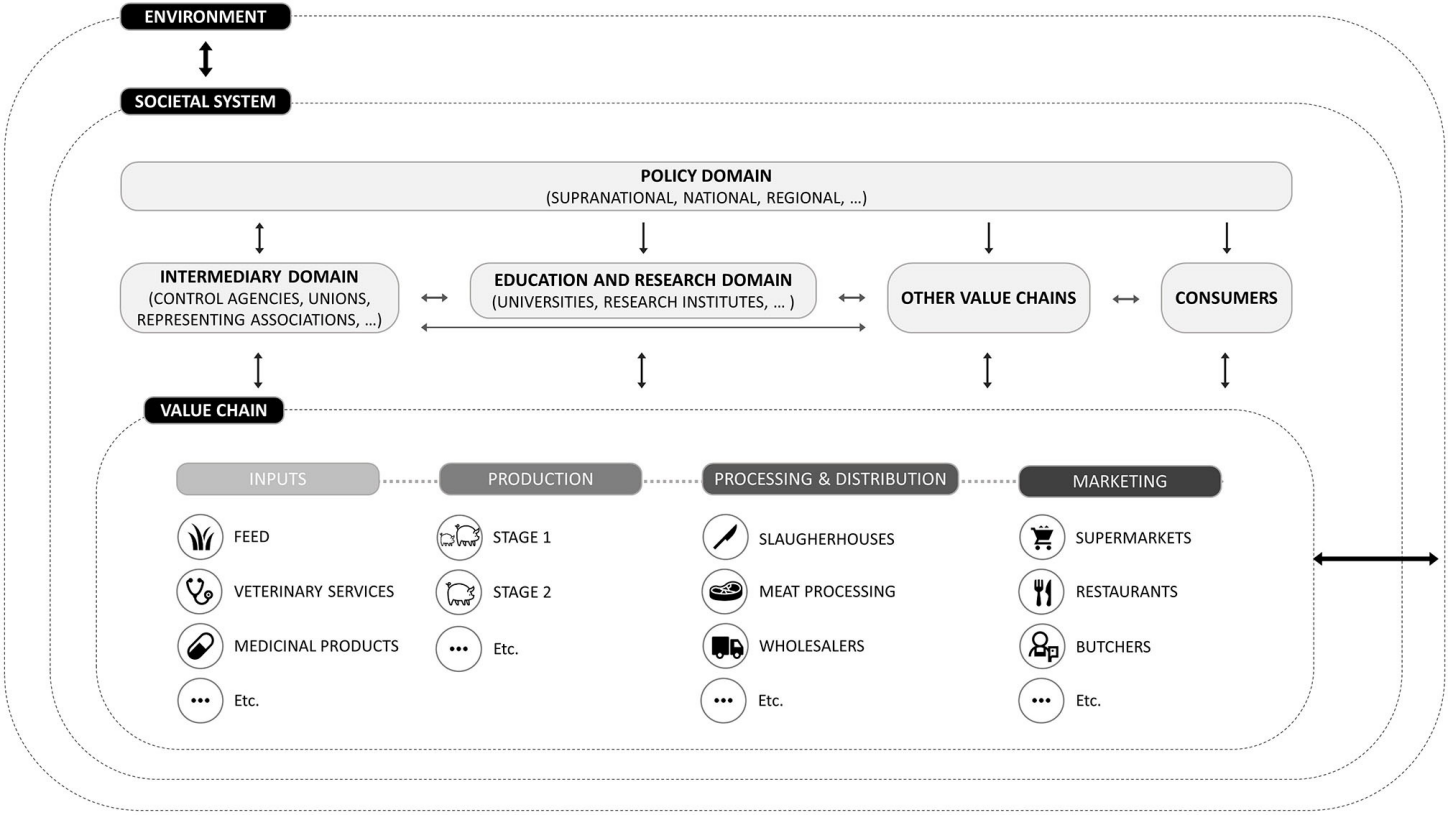
Interactions between value chains, societal systems and the environment.

To take a systemic look at antimicrobial use in livestock production, Figure 1 presents a simple representation of a value chain integrated in a bigger societal system and environment. Consistent with the literature on value chain analyses, the links of the chain have been divided into 4 categories: inputs, production, processing and distribution, and marketing (42, 43). In the context of livestock production, inputs refer to all the goods and services that are needed to raise livestock such as veterinary services, veterinary medicinal products, and feed. Production refers to the farming of the animals and can, depending on the production system, include several stages. For example, weaner producers can be specialized in breeding piglets, which will subsequently be sold to a fattening farm where the pig production cycle will be finalized. The processing and distribution category involves the slaughtering of the animals as well as the further processing of the meat. Finally, distributors such as retail, food suppliers, restaurants and exports are labeled as distribution and marketing.

When considering the value chain, it becomes more clear that besides farmers and veterinarians, other actors of the value chain could be targeted. In the upstream part of the value chain, input suppliers such as feed mills could be subject to policies, e.g., by banning or further regulating the production of certain inputs like non-medicated feed to avoid cross-contamination with antimicrobial residues (44, 45). Targetable actors can also be found in the downstream part of the chain, as, for example, the knowledge of truck drivers regarding the health of animals for transportation could be regulated (46) and the compliance of transportation companies to strict rules regarding the cleaning and disinfection of lorries could be controlled (47). Lately, labeling systems have also been set up to provide information about the antimicrobial use during the production of animal products (48).

When looking at the societal system in which a value chain is integrated, the actors it comprises may also influence actors and practices in the value chain. In Figure 1, these actors external to the value chain were divided into four categories from which the first three were based on Rojo Gimeno et al.'s (37) framework to characterize animal health: a policy domain, an intermediary domain, an education and research domain, and consumers. The policy domain comprises several levels such as (supra)national and regional governments. The intermediary domains refers to actors that, on the one hand, advise governments and may perform governmental activities and, on the other hand, may influence the value chain as well as the research and education domain via collaborations and the development of awareness campaigns. Finally, the research and education domain comprises schools, research institutions and universities developing and providing knowledge for the other actors as well as private and public extension organizations.

Such external actors could also be targeted by policies by for example subsidizing control agencies or farmers' organizations to develop communication campaigns to raise awareness. Universities could be expected to improve courses on AMR in the curriculum of veterinarians or farmers (49) or could be compensated for it. Educational campaigns about AMR, antimicrobial stewardship or biosafety could be promoted for farmers (50), veterinarians (51) and also advisors (from, e.g., feed mills or companies that work on farm equipment) (52). Such campaigns can also be supported by industries, such as pharmaceutical companies (53). Investment in R&D could allow the development of new tools. Alternatively, Giubilini et al. (54) suggest taxing meat, allowing consumers to compensate society for the AMR they contribute to by consuming meat that was produced with the use of antibiotics.

### Defining a Basis for Policies and Strategies

In this section, we will explore different options that may provide an optimal basis for measuring impact, or in other words, a variable that policies and strategies are intended to change. To serve as an optimal base to formulate a regulation or strategy and to measure compliance with that regulation/strategy, any elements in the input/technology-production and AMR relationship can be used as long as they are (a) correlated with AMR; (b) enforceable; and (c) targetable in space and time (33).

**Figure 2:**
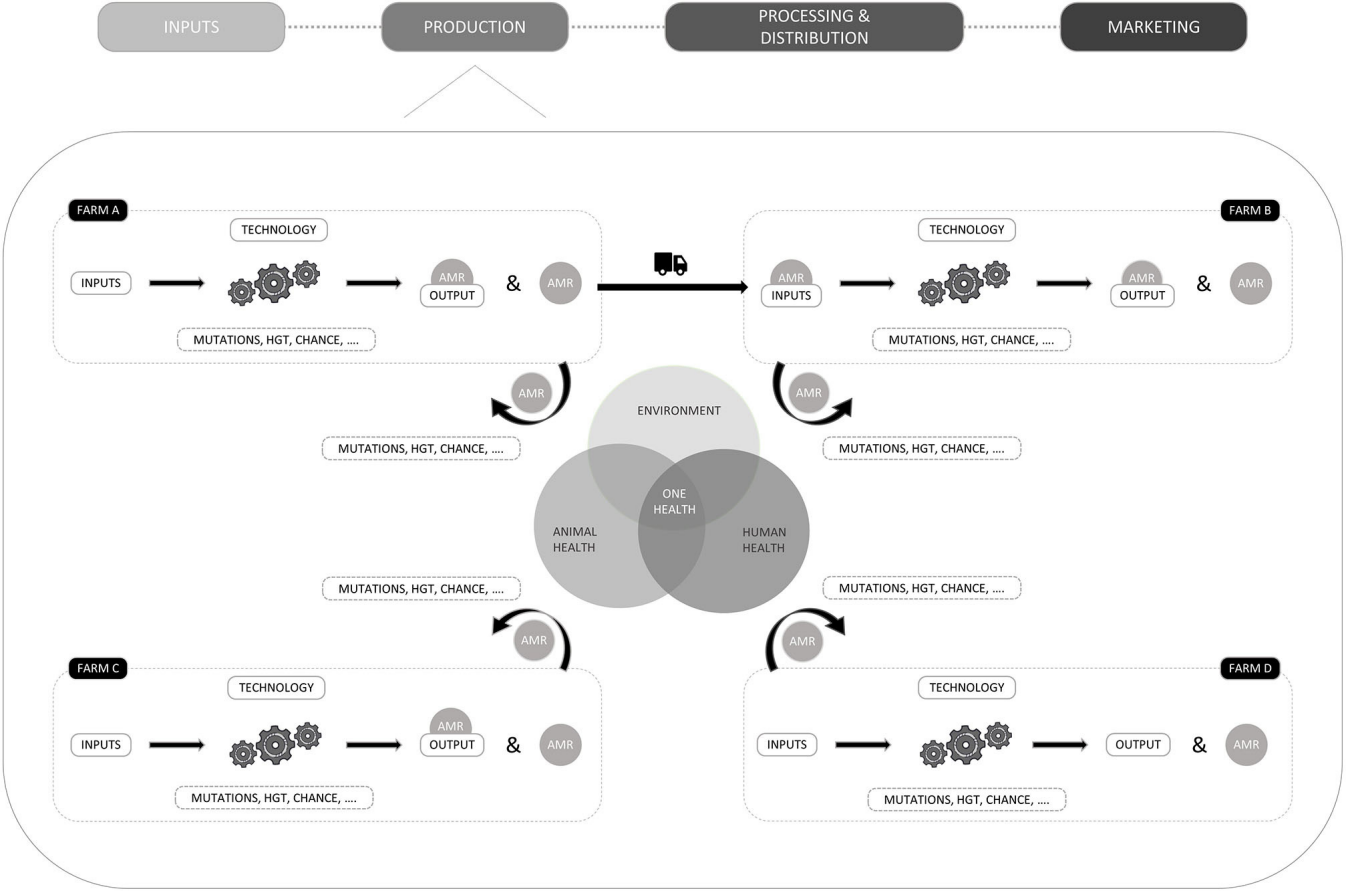
Graphical representation of the potential interaction of farms within a value chain with their environment and with each other. The potential contribution of four farms to AMR and the interactions with their environments is provided.

To identify several bases that could be used, we systemically analyzed the production segment of a livestock value chain. This exercise may of course be expanded to other segments of a value chain or layers of a system, as done by the UK government who produced AMR systems map to provide an overview of the factors influencing the development of antimicrobial resistance and the interactions between them in a one health context (55). The decision to restrict the analysis on actors and pathways regarding AMR in this paper relies on our aim to illustrate how systemic approaches may contribute to the identification of compliance bases rather than providing an extended overview of the ways in which a system may contribute to AMR. To this end, Figure 2 pictures the potential contribution of four farms (A, B, C, D) to AMR. For every farm, livestock production is presented as a result of inputs and technologies. Inputs refer to the goods and services necessary for production. Technology, refers to the production system or methods used for animal production and can determine the choice of inputs, as some technologies require more inputs of one type and fewer inputs of another. An example is antibiotic free vs. conventional production, where disease prevention through enhanced biosecurity and vaccination is preferred to treatment with antimicrobial substances. Different combinations of inputs and technology are therefore leading to varying levels of livestock production (output) and AMR, which will also be influenced by natural variability due to favorable mutations in microorganisms, horizontal gene transfer, chance and others. When considering technology or production practices as a compliance base, biosecurity factors (56) or organic production (57) make good candidates as correlations with AMR have been demonstrated. There have been clear links shown between the quantity of AMU on the one hand, and AMR on the other.

It is important to note that, since a production cycle can be composed of several stages, inputs can also refer to animals, which can also influence AMR levels. This is represented in Figure 2, where the output of farm A is sold and transported to farm B, where it is considered an input. In case, the animals carry resistant microorganisms (pathogens or commensals that carry resistance genes), AMR can be introduced on farm B through the input and influence the prevalence of AMR. Such transfers of AMR between farms are also prone to natural variability and have been documented for, i.e., methicillin-resistant *Staphylococcus aureus* (MRSA) in pig farms in Norway (58) and for ceftiofur resistant *Escherichia coli* in Belgian broilers, where the hatchery of origin proved to be an important risk factor (59). Moreover, it is suggested that animals can be infected with resistant microorganisms in transport trucks (60). In Denmark, the Specific Pathogen Free system (SPF system), developed by the pig sector in collaboration with universities, aims to avoid the introduction of new pathogens into pig herds via strict biosecurity rules, health control and transportation of pigs between herds. The system comprises 75% of the pigs born in Denmark and herd held statuses are publicly available (17).

Besides animals, the use of inputs such as antimicrobials have been proven to influence AMR levels on farms (7) and are therefore a good base to formulate a regulation. In, for instance, Denmark, the Netherlands and Belgium, antimicrobial consumption is monitored at farm level for several species, which allows the benchmarking of farmers and/or veterinarians (61). But even if the farmer is responsible for the chosen inputs, some aspects may be beyond control, such as the cross-contamination of non-medicated feed with residues of antimicrobials in feed mills or transport trucks (45). In 2016, Filippitzi et al. (45) estimated that 5.5% of the total feed produced in a year could be cross-contaminated with different levels of antimicrobials when antimicrobial medicated feed represented 2% of the total annual feed produced in a country.

In addition to inputs, outputs may also be targeted. Targetable outputs may include the type of animals that are produced (e.g., species and age) since different animal species metabolize drugs differently or the amount of output as consuming less animals could also reduce the use of antimicrobials (62). However, the correlation with AMR is less straightforward for these options than for input or technology based ones, which might therefore be preferred.

Finally, we should keep in mind that AMR can also be introduced in a farm through interactions with the environment or the ‘outside world’. Examples of such interactions include mutual use of farm workers or veterinary practitioners that can introduce resistant microorganisms (58), delivery trucks that travel from one farm to another (63), pests like rats (64), insects (65), or antimicrobial residues in the environment (66). When considering the One Health approach, antimicrobial residues from human wastes may end up in the environment (66) and subsequently influence AMR levels on farms. The level of interaction with the outside world is also influenced by technology, as, for example, free-range animals interact more with the environment than intensively produced animals that remain in stables.

With regard to interactions between farms and the ‘outside world’, it might be more complicated to find compliance bases. In such cases, AMR proxies such as monitored AMR trends could be used. Such options have a higher correlation with AMR but are less attributable to a producer than input/technology and production bases. Moreover, the surveillance of AMR trends in the European animal production is currently limited and results are published with a 2-year delay. To compensate, national surveillance systems have been put in place, each with their own sampling, testing and reporting modalities, yielding results that cannot be compared (67).

### Taxonomy of AMR Interventions: Five Routes to Behavioral Change

**Figure 3:**
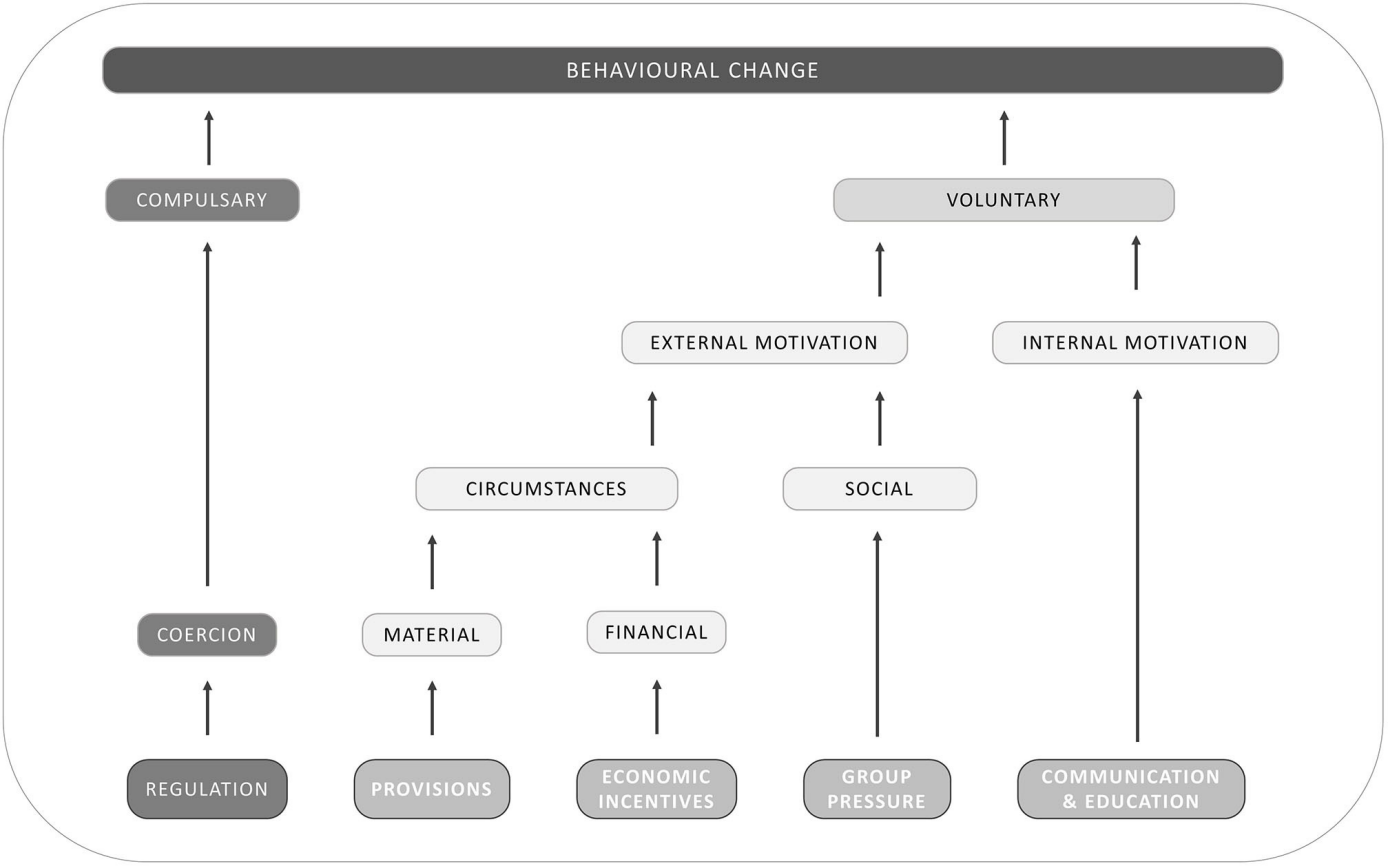
Possible routes to induce behavioral change [adapted from Van Woerkum, published in Leeuwis (69)].

Once it is defined who and what should be targeted, the next step is to determine the mechanisms through which the intended actor(s) and variable(s) can be targeted. This entails the choice for a policy instrument, an advisory approach, a structural intervention and other types of options. To change behavior, several intervention frameworks have been developed for different contexts such as policy making and retail (68). While the used vocabulary or level of detail regarding the categorization of the intervention type may differ, most of these frameworks consider regulation and coercion, norms, social influence and networks, knowledge, incentivization and enablement as factors that may influence one's behavior (68). Our choice was for Van Woerkum's exhaustive classification of interventions into five possible routes (69) (Figure 3) since it has recently been adapted and applied in veterinary sciences (70, 71).

According to Van Woerkum's framework, a first way to achieve behavioral change involves regulation (69). This route differs from the others in that it attempts to make change compulsory, in contrast to the others that strive to induce voluntary change. Therefore, “bad” behavior is made illegal while “good” behavior is made mandatory. The best known regulations on antibiotics are the ban on the use of antimicrobials as growth promoters in the EU. and the yellow card policy in Danish pig farming (36). Moreover, regulations can also be organized at sector level as part of quality systems, as was the case for Dutch veal calves, broilers and pigs (72).

To induce a voluntary change, the second route includes provisions and tools, which are instruments that are implemented to change the external material circumstances so that people become motivated to change their behavior. In some cases, the provisions can be restrictive (by making the ‘bad’ behavior less straightforward) and behavioral change is then coerced. However, in the case of AMR and AMU, most provisions and tools are rather enabling and give the intended person external motivation to voluntarily change by making it easier and more achievable to reduce and improve the use of antimicrobials. Examples of tools include coaching sessions to develop and implement farm health plans (73) and alternatives treatments such as bacteriophage therapy (74).

Another way to change external circumstances involves the use of economic and financial incentives. Here, the attempt is to create external circumstances that change the financial conditions in such a way that behavioral change is favored. Typical examples include subsidies for the ‘good’ behavior or taxes (fines) for the “bad” behavior. In the European Union, several countries have levied a tax on the sale of antibiotics (75, 76). Private companies can also use this approach by paying a price premium for products that are produced in ‘good’ production systems through labeling of products as produced without the use of antibiotics (77).

In addition, a third and more direct method to change external motivation of decision makers relates to group pressure and social norms. This mechanism attempts to induce behavioral change by making use of the typical desire of people to comply with the group norm. It is the attempt to use existing social norms, and make people aware of these norms. In some cases, new social norms need to be formed prior to this, if the social norms within the target group does not support the intended behavioral change. An example for the use of group pressure is the benchmarking of farmers and/or veterinarians based on AMU in Belgium, Denmark and the Netherlands (61).

Finally, the last route to voluntary behavioral change goes through communication and education. Through these mechanisms, change agents attempt to change the internal motivation of decision makers so that they become convinced that behavioral change is the best decision. Newly developed tools or economic studies that demonstrate the cost-effectiveness of measure to reduces AMU, such as improved management strategies (i.e., biosecurity strategies) (78) can be used as incentives. Within this category, typical extension instruments such as articles in agricultural magazines, demonstration farms, leaflets, study days, digital apps, and others are found. This is arguably one of the most used and investigated routes, mainly in the field of social veterinary epidemiology, which is the study of human behavior that affects the causes, spread, prevention and control of animal diseases and health problems (79), and related disciplines (80–82).

## Design and Evaluation of Policies and Strategies: Evaluation Criteria

The design of policies and strategies to reduce antimicrobials resistance is essentially guided by three criteria, being effectiveness, efficiency, and fairness (equity). These criteria should be used when evaluating, both ex-ante and ex-post, the performance of policies and strategies. Effectiveness refers to the question whether the implemented policy or strategy achieves its goal, i.e., a reduction in AMR. Policies or strategies that are not effective should not be further considered. In a world with unlimited resources, effectiveness would in fact be the only criterion of importance. However, this is not the case, especially for financial resources and time.

All policies and strategies within each of the five behavioral routes involve the allocation of resources. At farm level, policies and strategies usually aim to change AMU, biosecurity and production practices in general and/or to stimulate the adoption of alternative disease management measures. All this involves costs and benefits of which the end result may be negative or positive. Producers of antimicrobials may incur financial losses if the use of antimicrobials is drastically reduced. Policies and strategies such as the use of social norms, communication and education require the investment of financial resources and time by extension agents, researchers and other organizations. As resources are limited, the allocation of resources to one type of policy or strategy may come at the expense of another. Hence, resource allocation of animal health control in general and the reduction of antimicrobial resistance in particular, has to be informed by structured analyses (83, 84). Two criteria to evaluate these considerations are efficiency and fairness.

The economic efficiency of a policy intervention is the greatest when the social benefits net of social costs are maximized, regardless of how these may be distributed (85). The two most common approaches to evaluate economic efficiency in animal health are cost-benefit analysis (CBA) and cost-effectiveness analysis (CEA) (84). The former assesses monetary values to costs and outcomes to compare the net benefits of different courses of actions. Whereas it is the preferred approach by economists, it has a number of difficulties, particularly the problem of assigning monetary values to impacts such as improved human health or reduced AMR. This problem is circumvented in CEA by comparing costs in monetary units to outcomes expressed in more technical units, e.g., reduction in AMR or percentage reduction in average AMU. Through CEA, the effectiveness of different policies and strategies can be compared according to their costs. Whereas it is not always feasible to formally apply a CEA framework in quantitative terms, due to, amongst others, data scarcity and uncertainty, the use of estimates and sensitivity analysis to accommodate for uncertainty in a more broadly defined cost-effectiveness way of thinking, can aid in setting and prioritizing policies and strategies (84, 86, 87). Currently, economic tools and even economic thinking is insufficiently represented in the animal health domain and is often applied to individual farm decision support, but not to programs aimed at improving animal husbandry with regard to the reduction of externalities (88). This can lead to inefficient policies and strategies and low value for (public) money.

One shortcoming of cost-effectiveness analysis or CBA is that they do not consider the distribution of costs and benefits across all actors involved. Important criteria related to this are equity and fairness. While both concepts are related, they are not identical. Equity refers to the mere distribution of costs and benefits of different policies and strategies. Typically, these are not equally distributed over society, especially with an issue like AMR. Whereas the benefits are usually for society at large, through a decreased human health burden, costs often accrue to one or more specific groups, such as farmers, veterinarians or pharmaceutical companies. Nonetheless, the distribution of costs and/or benefits between different regions or groups may be a considerable measure when choosing between different policy options. Likewise, unequal distribution of costs and benefit can give rise to substantial opposition against new policies or strategies and may be the source of considerable lobby group efforts to weaken more severe regulation.

A problem is that equity considerations have been very resistant to rigorous analytical treatment. One of the reasons is the many competing notions of equity, which makes that the concept is analytically slippery. This becomes more clear when the notion of equity is replaced by the related concept of fairness. Whereas equity considers the mere distribution of costs and benefits across society, the concept of fairness refers to whether that distribution is socially just and acceptable. Fairness is a concept with which analysts are not comfortable, because it is open to subjective evaluation. One principle to overcome some of this difficulty is the Polluter Pays Principle (PPP), an environmental policy principle which requires that the costs of pollution be borne by those who cause it. In its original emergence, the PPP determined that the costs of pollution prevention and control must be allocated to the polluter. Its immediate goal is that of internalizing the environmental externalities of economic activities, so that the prices of goods and services fully reflect the costs of production (89).

Another concept related to this is the political feasibility of options if dealing with options that have to be decided upon and set by the government, such as public standards and taxes (90). Even if an instrument is theoretically effective and efficient, it can never be effective in practice if it is shot down in the political decision process. A proposed method to improve the political feasibility of policies and strategies is participatory design, i.e., the involvement of actors and stakeholders in the setting and prioritization of policies and strategies. Above the fact that such approaches might lead to more relevant, effective, and technically feasible measures, they have the advantage that participation of all involved and affected stakeholders in the design of the measures could lead to higher acceptability and thus higher political feasibility, which in turn leads to higher effectiveness.

## Discussion

**Figure 4:**
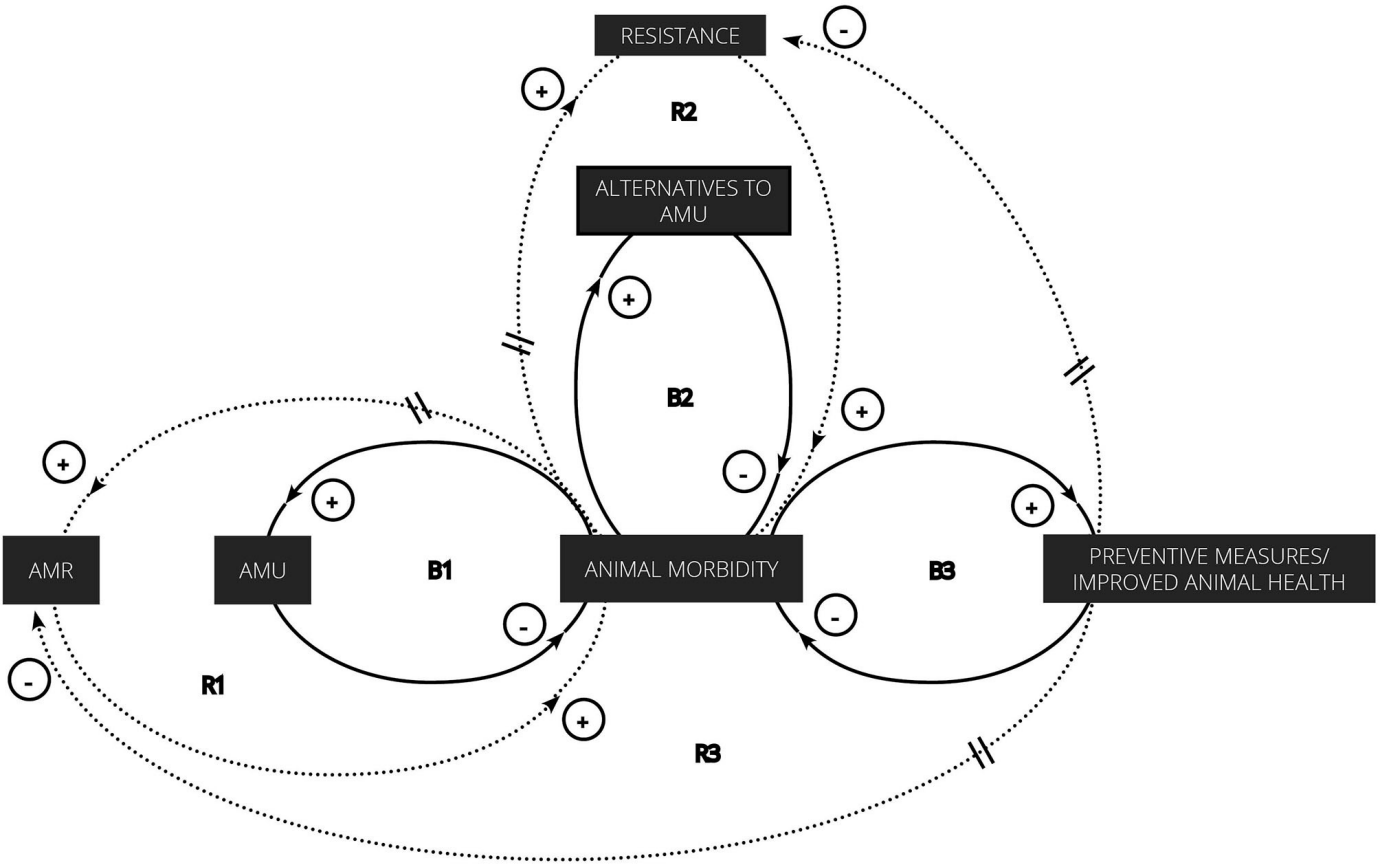
“Fixes that fail” archetype adapted to represent the interconnection between AMU, therapeutic alternatives to antimicrobials, preventive measures/improved animal health, resistances, and animal morbidity in livestock production.

In our introduction, we discussed the global political will to contain AMR through surveillance and optimal antimicrobial use and prescription. For the latter two, strategies are focused on reducing use and dependence, often without taking the behavioral character of AMU and AMR into consideration. To better visualize the interconnection between human decisions concerning AMU and AMR in livestock production, we looked at it from a systems perspective and adapted “the fix that fails” system archetype to represent the relationship between AMR and AMU. System archetypes are causal loop diagrams—or visual representations of balancing (B) and reinforcing (R) processes in a system—that seem to recur in many different life settings. The “fixes that fail” archetype involves the quick implementation of a solution to alleviate symptoms (91). The relief is however of short duration since unintended consequences arise from the solution over a long period of time or as an accumulated consequence of repeatedly applying the solution (91). In our system of interest, antimicrobials are used to treat animal morbidity, thus decreasing or balancing the latter (see B1 in Figure 4). Unfortunately, this repeated use leads to a delayed and unintended increase in AMR prevalence, which in turn reinforces animal morbidity (R1 in Figure 4). Along with antibiotics, alternatives to antibiotics such as phage therapy (B2 in Figure 4) and preventive measures/an improved animal health (B3 in Figure 4) can also balance animal morbidity. The first option will, however, suffer the same fate as antimicrobials since repeated use will result in resistance to these therapies (R2 in Figure 4). Only the improvement of animal health and disease prevention, which involves structural changes rather than quick solutions, is therefore expected to balance the prevalence of resistance in addition to animal morbidity.

This simple representation of the interrelationships between animal morbidity, solutions and consequences allows to easily visualize the imbalance that has been created by repeatedly reinforcing the same feedback loop as well as the unintended consequence that results from it. In order to solve this, global efforts are being made to contain AMR, mainly by institutionalizing AMU and by trying to reduce the dependence on AMU, the former acting on the first balancing loop (B1) in Figure 4 and the latter on the two other balancing processes (B2 and B3). Since everything is interconnected, interventions in one feedback loop may also impact the others. For example, an improved animal health is expected to decrease animal morbidity, which, in turn, should result in a decreased need for antimicrobials and alternatives thereof, both of which have proven to be extremely valuable and indispensable assets for animal and human health. By juggling the use of both, the prevalence of corresponding resistances may be kept to levels that will not menace public and animal health, stabilizing their efficacy to decrease animal morbidity.

Addressing AMR in a sustainable manner is therefore based on the development of strategies and measures that aim to achieve an equilibrium where animal morbidity is reduced through solutions that enhance/preserve animal health, antimicrobials and their alternatives without compromising the effectiveness of the latter two. The effectiveness of these strategies and policies relies on whether these are technically, economically, behaviorally, and politically feasible in a certain temporal and spatial context.

In order to develop such strategies and policies, we addressed three fundamental questions on which control policies and strategies for agricultural pollution problems are centered in the light of AMR (34). We demonstrated the usefulness of systemic approaches to define who, what and how to target by considering the complexity in which the ultimate decision-maker is embedded. With regard to the third question, we explored five routes for behavioral change, being regulation, provisions, economic incentives, group pressure and communication and education. Whereas, this scheme has been developed in environmental sciences and economics to describe options to stimulate behavioral change regarding land use management and environmental sustainability, it has recently been popularized in veterinary sciences by Wessels et al. as the R.E.S.E.T. model (70) and was used by Lam et al. (71) to showcase interventions to change the antibiotics use behavior of Dutch dairy farmers with the suggestion that all the routes should be used in order to reach the entire sector. While this scheme was aimed at individuals, we also believe that it can be used to categorize AMR interventions at system level, in this case livestock production. A clear taxonomy moreover allows for the identification of gaps in current strategies, as this was done for antibiotic stewardship in human medicine (92). Moreover, we also believe that every way of targeting actors should not be used to the same extent in order to effectively manage AMR with limited resources. In this regard, we propose to guide the design of policies and strategies to contain antimicrobials resistance by considering their effectiveness, economic efficiency, and fairness (equity) and by defining cost-effective combinations of instruments within the different categories to target the relevant actors of a system. These criteria are seldom being used in the design of policies and strategies for AMR and even less so in a holistic and systemic sense, that is, taking into account the full breadth of potential actors and bases as well as the full breadth of potential policies and strategies across all five routes of behavioral change. This is exacerbated by the fact that policies and strategies within the five behavioral change routes are often investigated within different and distinct disciplines. Researchers in veterinary medicine and veterinary epidemiology mainly deal with technical measures that will result in a reduction in the use of antimicrobials, such as better biosecurity, vaccination and alternatives to antimicrobial, which are sometimes hard to adopt due to a lack of social science knowledge. More recently, the field of social veterinary epidemiology, which is composed of research from both veterinary epidemiology and social sciences, has boomed, resulting in an amplification of studies that aim to inform policies and strategies mainly from within the communication and education route. Finally, economists are often mainly dealing with economic incentives such as taxes and labels, and to a lesser extent with regulation, most often private standards.

These observations, in addition to the systemic approaches we are suggesting to use to define who and what to target, point to a need for more interdisciplinary research. The final decision of how and when to use antimicrobials has a non-linear, uncertain and unpredictable character that is shaped by various factors that are inherent to a system. Such a system is best understood when tools and methods from different disciplines are used. Such approaches, here referred to as interdisciplinary systemic approaches, have already been documented in relation with urban (93) and agricultural (30) sustainable solutions for climate change, and sustainable energy use in homes (94). They have also been advocated for in the context of antimicrobial resistance by Flowers (95), who considers that public health policies lack a systems perspective and highlights the added value of health psychology, that focuses on the individual, and its synergies with medical sociology, which focuses on the systems and organizations. In addition to crossing the boundaries of disciplines, we suggest to go a step further by advocating for the involvement of stakeholders in the development of solutions and strategies *via* participatory approaches, which could be considered by some as a shift from interdisciplinarity to transdisciplinarity (96). According to Stock and Burton (97), transdisciplinarity mainly differs from interdisciplinarity by aiming to synthesize new disciplines and theory. In the case of antimicrobial resistance, the One Health approach is often referred to as inter- and transdisciplinary, while the degree of integration in practice varies between, on the one hand, improving knowledge exchange and communication between environmental, animal health, and public health research and, on the other hand, truly viewing these domains as interconnected and therefore as one research area. However, the contributions of the different disciplines within One Health often remain very discrete, meaning that problems that are addressed in a One Health way, are addressed from the perspective of one discipline across the three research domains. For example, topics that are studied in both human and animal health are often approached from a biotechnological perspective, leaving out social and economic sciences. In contrast with this, our approach aims to promote a true holistic perspective based on goal-oriented interdisciplinary research. This relates to having a strong Agricultural Knowledge and Innovation System (AKIS) (98) and veterinary public health sector, with a critical mass of diverse stakeholders that collaborate in concerted efforts. The stakeholder mass should encompass both business and transdisciplinary actors (e.g., farmers, companies) and non-business actors such as organizations (e.g., farmers' organizations, federations of veterinarians) and public institutes and organizations. These stakeholders should collectively strive for such a goal-oriented interdisciplinary agenda. Moreover, the public institutes and organizations such as public animal and/or human health agencies could take the lead to facilitate this. For example, Living Labs could be set up as part of research and innovation projects (99) and knowledge centers funded by public-private partnerships could be created to steer this process.

In addition to obtaining a holistic view of a problem and identifying solutions, interdisciplinary systemic approaches can further allow to anticipate unexpected positive or negative side effects that the solutions may entail. For example, reducing AMU to solve AMR will also help reducing AMR pollution that could disturb ecosystems (66), but might have negative implications when it comes to animal welfare.

Finally, in addition to the defining who, what and how to target *via* interdisciplinary approaches, we identified a fourth question that relates to who will implement the regulation or strategy, i.e., by whom the party of interest will be targeted. Indeed, when considering policies or strategies, it is often considered that these will be implemented by the government. However, key actors in livestock production systems could be used to target the party of interest. For example, farmers' unions or farming schools could develop courses on biosecurity to incite farmers to improve this on their farms. Consumers could be sensitized to the problem *via* retailers by obtaining information about the use of antimicrobials during the production of the meat. In this regard, some companies are currently trying to provide their customers with information about the production and provenance of their products *via* the block chain technology in an attempt to enhance transparency regarding food supply chains (100).

## Conclusion

Strategies and policies that focus on reducing use and dependence to antimicrobials often do not take the behavioral character of AMU and AMR into consideration. To address this, we have introduced an approach that relies on interdisciplinary systemic approaches to comprehensively characterize antimicrobial decision system, hence identifying all actors influencing AMU in livestock production, adequate regulatory and intervention bases, which behavioral change strategies to use and whom should implement this. In addition, we suggested to identify the best combinations of behavioral strategies through cost-effective analyses since economic and time resources are limited. To enable the development of policies and strategies *via* the suggested approach, several areas for further research arise:

- Interdisciplinary systemic research to assess the behavioral aspect of AMR by characterizing antimicrobial decision systems in livestock production systems.
- Interdisciplinary research allowing the development of solutions or interventions across several disciplines in order to enhance their overall feasibility.
- The use of participatory design (or co-creation) approaches in order to develop solutions that are adapted to the context in which decision-makers are embedded.
- Economic analyses in order to identify cost-effective combinations of interventions from different behavioral change routes at system level.

## Author Contributions

The writing was mainly performed by FB and EW. All authors contributed equally to the conceptual thinking and editing of this paper.

## Conflict of Interest

The authors declare that the research was conducted in the absence of any commercial or financial relationships that could be construed as a potential conflict of interest.
